# A Wearable-Derived Autonomic Component Is Associated with Cardiovascular Health in Community-Dwelling Adults: A Cross-Sectional Analysis from the Longevity Check-Up 8+ Study

**DOI:** 10.3390/medsci14050540

**Published:** 2026-09-02

**Authors:** Riccardo Calvani, Fabrizio Sollazzo, Luca Maria Sarti, Antonella Gallo, Anna Picca, Hélio José Coelho-Júnior, Andrea Russo, Sara Salini, Massimiliano Bianco, Emanuele Marzetti, Vincenzo Palmieri, Francesco Landi, Matteo Tosato

**Affiliations:** 1 Department of Geriatrics, Orthopedics and Rheumatology, Università Cattolica del Sacro Cuore, 00168 Rome, Italy; riccardo.calvani@unicatt.it (R.C.); lucamaria.sarti01@icatt.it (L.M.S.); coelhojunior@hotmail.com.br (H.J.C.-J.); francesco.landi@unicatt.it (F.L.); matteo.tosato@unicatt.it (M.T.); 2 Department of Geriatrics, Orthopedics and Rheumatology, Fondazione Policlinico Universitario Agostino Gemelli IRCCS, 00168 Rome, Italy; antonella.gallo@policlinicogemelli.it (A.G.); picca@lum.it (A.P.); andrea.russo1@policlinicogemelli.it (A.R.); sara.salini@policlinicogemelli.it (S.S.); 3 Sports Medicine Unit, Fondazione Policlinico Universitario Agostino Gemelli IRCCS, 00168 Rome, Italy; fabrizio.sollazzo@unicatt.it (F.S.); massimiliano.bianco@unicatt.it (M.B.); vincenzo.palmieri@unicatt.it (V.P.); 4 Scuola di Specializzazione in Medicina dello Sport e dell’Esercizio Fisico, Università Cattolica del Sacro Cuore, 00168 Rome, Italy; 5 Department of Medicine and Surgery, Libera Università Mediterranea (LUM) Giuseppe Degennaro, 70010 Casamassima, Italy

**Keywords:** heart rate variability, physical activity, sleep, preventive medicine, wearable devices, digital medicine, glycaemic health

## Abstract

**Background/Objectives**: Wearable devices provide multidimensional measures of cardiometabolic health. We aimed to derive latent physiological dimensions from ECG and motion variables collected by a wearable device and evaluate their associations with the individual health metrics of the Longevity Check-Up 8+ (LC8+) programme. **Methods**: Community-dwelling adults voluntarily participating in four Longevity Check-Up 8+ (LC8+) events underwent 24-h multiparameter recording using a wearable device. Eight wearable-derived variables were entered into principal component analysis (PCA) to derive latent physiological dimensions. Component scores were subsequently evaluated for associations with overall LC8+ health, while exploratory analyses examined associations with individual health metric domains. **Results**: Ninety-seven participants (mean age 51.3 ± 13.4 years; 36% female) were enrolled. Among the 96 participants with complete data for the PCA, three latent physiological components were identified, explaining 70.1% of the total variance. PC1, which was characterised by high SDNN, high RMSSD, and low nocturnal heart rate, was independently associated with overall LC8+ health (LM: B = +0.402, 95% CI [+0.103, +0.700], *p* = 0.009). Among the eight LC8+ domains, only the association between PC1 and glycaemic health remained statistically significant after Benjamini–Hochberg correction in the primary fixed-effects analysis (95% CI [1.32, 4.62], adjusted *p* = 0.039). **Conclusions**: A wearable-derived autonomic component was associated with overall cardiometabolic health in community-dwelling adults. Among the individual LC8+ domains, the strongest association was observed for glycaemic health. These preliminary findings support further investigation of wearable device monitoring as a complementary tool for cardiovascular health assessment in preventive medicine.

## 1. Introduction

Wearable devices enable continuous, non-invasive monitoring of cardiovascular physiology outside traditional clinical settings [1]. The rich physiological information generated by these devices is increasingly recognised as a source of candidate digital biomarkers, providing objective and longitudinal measures of health that extend beyond conventional clinical assessments [2]. Beyond routine arrhythmia detection, these devices capture multiple physiological signals, such as heart rate variability (HRV), nocturnal heart rate dynamics, and sleep-related parameters, that may reflect complementary physiological domains relevant to cardiometabolic health [3,4].

Among the wearable-derived physiological measures, indices of autonomic function, particularly HRV, have consistently been associated with cardiovascular health [5], metabolic disorders [6,7], physical fitness [8] and healthy ageing [9], while sleep-related parameters have emerged as complementary markers of cardiometabolic risk [10].

The Longevity Check-Up 8+ (LC8+) initiative provides a multidimensional framework for assessing cardiovascular health in community-dwelling populations through eight health metric (HM) domains, including smoking, diet, physical activity, blood pressure, cholesterol, blood glucose, sleep quality and body mass index (BMI) [11,12].

Whether wearable-derived physiological signals reflect the overall cardiovascular health profile or preferentially map onto specific health domains remains unknown.

In this cross-sectional study, we characterised latent physiological patterns from wearable device recordings and investigated their associations with overall and individual cardiovascular health metrics within the LC8+ framework.

## 2. Materials and Methods

### 2.1. Study Design and Participants

This cross-sectional observational study included community-dwelling adults attending four public Longevity Check-Up 8+ (LC8+) preventive medicine initiatives held in Italy (Gaeta, Ovindoli, Rome Longevity Run, and Roma Urbs Mundi). Participants attending the events were given the opportunity to undergo 24-h wearable recording on a voluntary basis, subject to the number of Rooti Rx devices available at each event. Participation in wearable recording was therefore self-selected and capacity-limited by device availability. Participants who underwent wearable recording and provided informed consent were included in the study.

### 2.2. Longevity Check-Up 8+ (LC8+)

The Longevity Check-Up 8+ (LC8+) is an ongoing initiative of the Department of Geriatrics, Università Cattolica del Sacro Cuore, and Fondazione Policlinico Universitario “Agostino Gemelli” IRCCS, Rome, Italy, designed to promote cardiovascular health and healthy lifestyle behaviours in the general population [11,12]. LC8+ events are community-based preventive medicine initiatives conducted in non-clinical settings, including city squares, shopping centres, sporting and cultural events, and other public venues. The initiative is open to all adults and provides free cardiovascular health assessment and lifestyle counselling in community settings. The full protocol is described elsewhere [11,13].

Cardiovascular health was assessed using the Longevity Check-Up 8+ (LC8+) framework, an adaptation of the American Heart Association’s Life’s Essential 8 (LE8) construct for rapid community-based screening [14]. The LC8+ includes eight health metric (HM) domains: smoking, diet, physical activity, body mass index, blood pressure, blood cholesterol, blood glucose, and sleep quality. Unlike the graded 0–100 scoring system used in LE8, LC8+ applies simplified binary criteria adapted to rapid community-based assessment, with some operational thresholds being more permissive than the corresponding LE8 definitions. Lifestyle information was collected through a brief standardised interview, whereas body mass index, blood pressure, capillary blood glucose, and total cholesterol were measured on-site using standardised procedures [13] (Supplementary Table S1). Because screening was conducted in community settings, requiring participants to attend after an overnight fast was not feasible; capillary glucose was therefore measured irrespective of fasting status, which was recorded at the time of assessment. According to the prespecified LC8+ algorithm, the glycaemic health target was defined as capillary glucose < 100 mg/dL in fasting participants or <200 mg/dL in non-fasting participants, in both cases in the absence of glucose-lowering therapy. The non-fasting threshold was adopted as a pragmatic screening criterion informed by internationally established thresholds for marked hyperglycaemia on random glucose testing, rather than as a definition of optimal non-fasting glycaemia. Each domain was dichotomised as meeting (1 point) or not meeting (0 points) the predefined health target according to the LC8+ protocol, generating a composite HM Total score ranging from 0 to 8, with higher scores indicating better cardiovascular health. The rationale and operational definitions of the LC8+ assessment have been described previously [11]. The Ethics Committee of the Università Cattolica del Sacro Cuore approved the study protocol (#A.1220/CE/2011); all participants provided written informed consent.

### 2.3. Wearable ECG Monitoring: Rooti Rx Device

Participants wore the Rooti Rx device (Rooti Labs Ltd., Taipei, Taiwan), a lightweight wireless ECG patch providing continuous single-lead ECG monitoring for up to 7 days [15]. The device holds FDA 510(k) clearance (K163694; November 2017; 21 CFR 870.2910, Class II) and CE marking as a Class IIa medical device under the EU Medical Device Regulation (MDR 2017/745; Certificate TW24/00000573, SGS Belgium NV, Notified Body 1639; valid until July 2029), supporting its use for ambulatory ECG recording intended for interpretation by healthcare professionals. The device also incorporates a three-axis accelerometer, which provides motion signals that can be used to estimate chest wall movement during respiration. For the present study, participants were required to wear the device for at least 20 consecutive hours, including one complete nocturnal sleep period. The Rooti Rx device was fitted at the end of the LC8+ check-up, and participants wore it under free-living conditions, including their normal activities, before returning the device the following day. The device records ECG signals continuously at a sampling frequency of 250 Hz (24-bit analogue-to-digital resolution) and incorporates an integrated motion sensor that supports the automated analysis of sleep-related parameters. Raw ECG recordings were processed using the proprietary RootiCare analytical platform, which derives cardiovascular, sleep, and respiratory metrics from the ECG signal. Signal quality was monitored via an epoch-level effective rate: in each 60-s window, if the proportion of valid QRS intervals (noise pass rate) exceeded 75%, the epoch was classified as valid; HRV metrics were computed exclusively on valid epochs. Participants with a known history of atrial fibrillation or implanted cardiac pacemakers were excluded. In addition, all ECG recordings were independently reviewed by a sports medicine physician and a clinical cardiologist, who verified the absence of significant artefact, sustained arrhythmias, and pacemaker rhythms, providing a dual-layer quality control. Eight wearable-derived variables were extracted for analysis: (i) mean nocturnal heart rate (HR, bpm); (ii) SDNN (standard deviation of all NN intervals over 24 h, ms); (iii) RMSSD (root mean square of successive RR differences, ms); (iv) Cyclic Variation of Heart Rate Index (CVHRI, nocturnal cardiorespiratory events/h); (v) Chest Effort Index (CEI, respiratory effort events/h); (vi) sleep efficiency (%); (vii) wake after sleep onset (WASO, min); and (viii) mean sleep duration (h) (Supplementary Table S2). CVHRI and CEI are proprietary indices automatically derived by the Rooti Rx analysis algorithm for estimating nocturnal cardiorespiratory events and respiratory effort, respectively [16].

### 2.4. Assessments of Physical Activity

Because the LC8+ health metrics provide categorical (target achieved/not achieved) assessments of lifestyle behaviours, habitual physical activity was additionally assessed with the Godin–Shephard Leisure-Time Physical Activity Questionnaire [17,18]: total score = (vigorous sessions × 9) + (moderate × 5) + (light × 3); scores ≥ 24 indicate physically active status.

### 2.5. Statistical Analysis

Continuous variables are presented as mean ± standard deviation (SD), whereas categorical variables are reported as frequencies and percentages. Principal component analysis (PCA) was performed on complete cases for the eight wearable-derived variables. Variables were standardised (z-scores) within the analytic sample before PCA to identify latent physiological dimensions. Component retention was based on parallel analysis [19], supported by inspection of the scree plot, and component loadings were used to interpret the physiological meaning of each retained component. Sampling adequacy was assessed using the Kaiser–Meyer–Olkin (KMO) measure and Bartlett’s test of sphericity.

The primary association between wearable-derived dimensions and overall cardiometabolic health (HM total score) was evaluated using a linear regression model (LM) including PC1, PC2, PC3, age, sex, and recruitment site as fixed effects. Model assumptions were assessed by evaluating residual normality (Shapiro–Wilk test), homoscedasticity (Breusch–Pagan test), and multicollinearity using variance inflation factors (VIFs). Because only PC1 was significantly associated with the HM total score in the primary analysis, exploratory domain-specific analyses were subsequently performed for PC1. Each of the eight individual HM binary outcomes was analysed using a generalised linear model (GLM, binomial family, logit link) including PC1, age, sex, and recruitment site as fixed effects. This fixed-effect specification was designated as the primary model for the domain-specific analyses, given the limited number of recruitment sites (N = 4). Results are reported as odds ratios (ORs) with 95% confidence intervals (CIs). To control for multiple comparisons across eight domain-specific tests, the Benjamini–Hochberg (BH) false discovery rate correction was applied; BH-adjusted *p*-values are reported alongside nominal *p*-values.

Several sensitivity analyses were conducted to evaluate the robustness of the findings. First, the PCA was refitted after sequentially excluding variables with the lowest individual measures of sampling adequacy (MSA). These analyses were used to assess whether the component structure and the loadings of the principal wearable-derived components were sensitive to variables contributing least to the overall sampling adequacy. Second, analyses were repeated after excluding participants with extremely high values of heart rate variability metrics to evaluate the impact of potential outliers. Third, the primary association between PC1 and HM Total score was re-evaluated using an ordinal regression model, with HM Total score treated as an ordered categorical outcome and the same covariates included in the primary linear model. Fourth, the association between the wearable-derived components and HM Total score was re-evaluated using a linear mixed-effects model including recruitment site as a random intercept, while the domain-specific PC1 analyses were repeated using generalised linear mixed-effects models with the same random-intercept structure. Given the small number of recruitment sites, these mixed-effects models were considered sensitivity rather than primary analyses.

Additional sensitivity analyses addressing fasting status and glycaemic classification were performed for the primary overall-health model and for the HM Glucose domain-specific model, as detailed in the Supplementary Materials.

Given sparse site-by-outcome cells in some domain-specific models, Firth bias-reduced logistic regression was additionally used as a sensitivity analysis for the association between PC1 and the glycaemic health domain, retaining the same fixed-effect specification as the primary model; penalised profile-likelihood confidence intervals and *p*-values were used for inference.

Statistical significance was set at *p* < 0.05 (two-tailed). The analysis plan was not preregistered. All analyses were performed in RStudio (version 2026.05.0+18, Posit Software, PBC, Boston, MA, USA 2026) and jamovi (version 2.3.21.0, The jamovi project, Sydney, Australia, 2022).

## 3. Results

### 3.1. Participant Characteristics

Table 1 summarises the participant characteristics. The 97 participants were recruited across four LC8+ events: Gaeta (n = 30, 30.9%), Roma (n = 25, 25.8%), Ovindoli (n = 22, 22.7%), and Roma Urbs Mundi (n = 20, 20.6%). Mean age was 51.3 ± 13.4 years (range 22–77); 62 participants were male (63.9%) and 35 female (36.1%). The sex distribution varied across recruitment sites (e.g., the proportion of males was 80.0% in Gaeta, 36.0% in Rome, 68.2% in Ovindoli, and 70.0% at Roma Urbs Mundi). The mean Godin–Shephard score was 39.5 ± 21.7. Data were complete for most variables; CVHRI, CEI, and WASO were available for 96 out of 97 participants. For the LC8+ health metrics, the most frequently met criteria were physical activity (84.5%), sleep (80.4%), and BMI (75.3%); cholesterol was met by 32.0% of the participants. Mean HM Total score was 5.3 ± 1.5 (range 1–8) and was lowest at Roma Urbs Mundi (4.8 ± 1.2), partly reflecting a lower proportion of participants meeting the blood pressure criterion (20.0% vs. 46.7–68.0% at the other sites; Supplementary Table S3). Mean SDNN was 209.9 ± 55.1 ms and mean nocturnal HR 59.6 ± 8.2 bpm. The mean epoch-level effective rate was 98.2% (range 85.4–100.0%), indicating that nearly all 60-s ECG windows met the noise pass-rate criterion and were retained for HRV analysis.

### 3.2. PCA Structure of Wearable-Derived Variables

Of the 97 participants included in the study, one had missing data for CVHRI, CEI, and WASO and was therefore excluded from the complete-case PCA, resulting in an analytic sample of 96 participants.

**Figure 1:**
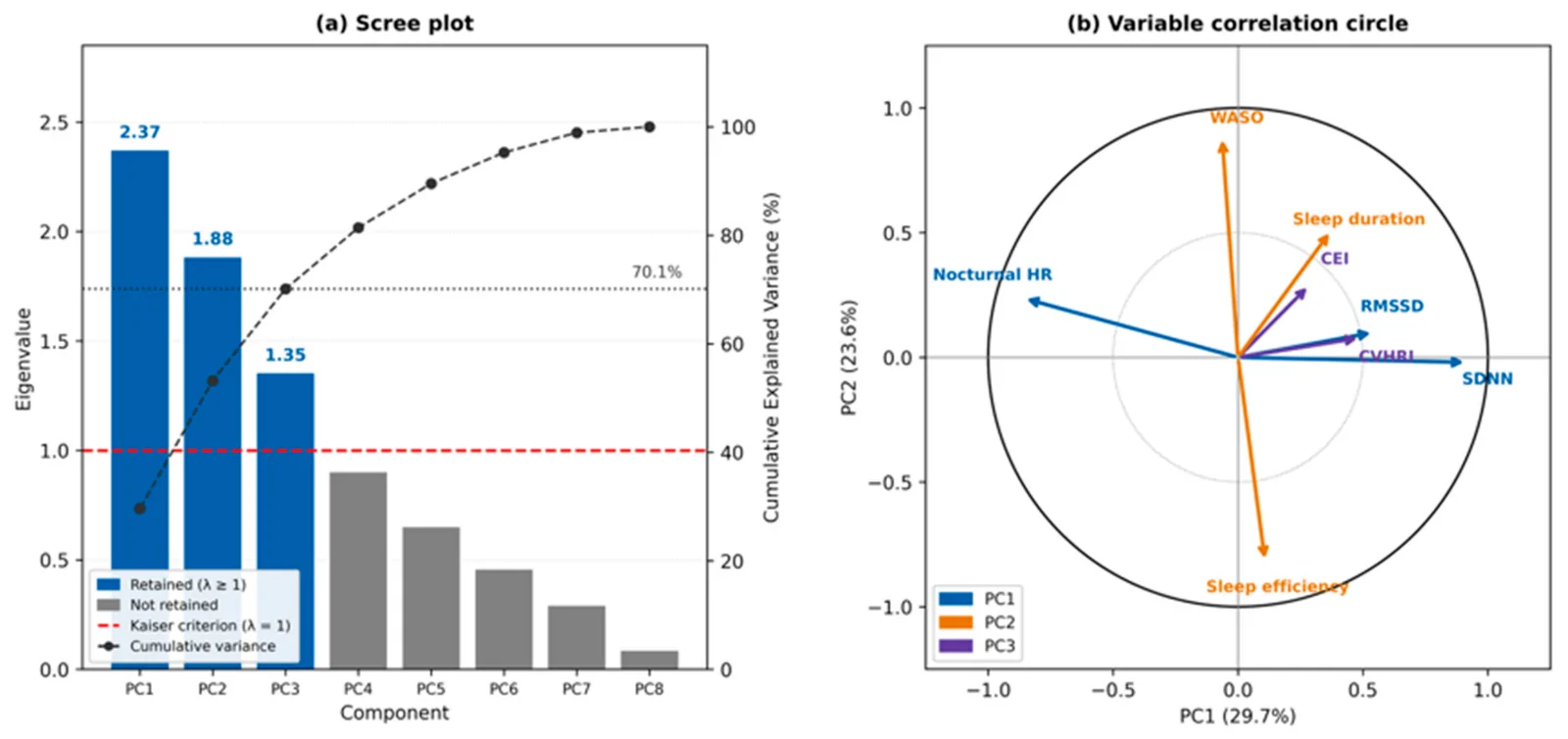
(**a**) Scree plot of eigenvalues for all eight components with cumulative explained variance (dotted curve = cumulative explained variance; cumulative variance after PC3 [dotted line] = 70.1%); parallel analysis retained three components (eigenvalues 2.37, 1.88, 1.35; all also satisfying the Kaiser criterion λ ≥ 1, i.e., each retained component explains at least as much variance as a single original variable). (**b**) Variable correlation circle (PC1 × PC2, unrotated): arrow length and direction indicate each variable’s loading on the first two principal components.

The overall KMO measure of sampling adequacy was 0.493, and Bartlett’s test of sphericity was significant (*p* < 0.001). Parallel analysis supported the retention of three principal components. The scree plot and Kaiser criterion (eigenvalues ≥ 1), shown in Figure 1a, were consistent with this three-component solution, which collectively explained 70.1% of the total variance (Table 2).

PC1 (29.7% of the total variance) was characterised by a strong positive loading for SDNN (+0.9205), a moderate positive loading for RMSSD (+0.5468), and a strong negative loading for nocturnal HR (−0.8623), indicating that higher PC1 scores corresponded to greater HRV and lower nocturnal heart rate. PC2 (23.6%) was dominated by WASO (+0.8876) and sleep efficiency (−0.8233), with a moderate positive loading for sleep duration (+0.5114). PC3 (16.9%) was driven by CVHRI (+0.6794) and CEI (+0.6747), with a moderate negative loading for RMSSD (−0.5781). The variable correlation circle (Figure 1b) shows the variable loadings on the first two principal components.

Individual measures of sampling adequacy (MSA) values are reported in Supplementary Table S4. CEI (MSA = 0.408) and RMSSD (MSA = 0.430) showed the lowest sampling adequacy. Therefore, sensitivity analyses were performed by sequentially excluding these variables. Although the exclusion of CEI alone and CEI plus RMSSD increased the overall KMO above the conventional threshold of 0.500, the overall component structure changed. Nevertheless, the variables defining PC1, particularly SDNN and mean nocturnal heart rate, retained similar loadings across these analyses (Supplementary Tables S5 and S6). In addition, the exclusion of participants with SDNN values > 300 ms (N = 7) yielded comparable PCA results, particularly for PC1 (Supplementary Table S7).

### 3.3. Association Between Wearable-Derived Physiological Dimensions and Overall LC8+ Health

A linear model including all three principal components, age, sex, and recruitment site as fixed effects showed that PC1 was the only wearable-derived component independently associated with HM Total score (B = +0.402, 95% CI [+0.103, +0.700], *p* = 0.009; Table 3). PC2 (B = −0.162, *p* = 0.278) and PC3 (B = −0.027, *p* = 0.875) were not independently associated with overall LC8+ health score. Age (B = −0.026, *p* = 0.037) and male sex (B = −0.668, *p* = 0.043) were also significant fixed effects.

Model diagnostics supported the adequacy of the linear regression model, with no major violations of model assumptions (Supplementary Figure S1).

Sensitivity analyses were consistent with the primary finding. The linear mixed-effects model with recruitment site as a random intercept yielded a similar association for PC1 (B = +0.408, 95% CI [+0.114, +0.702], *p* = 0.008; Supplementary Table S8). After excluding participants with SDNN > 300 ms, PC1 remained significantly associated with HM Total (B = +0.410, 95% CI [+0.052, +0.767], *p* = 0.025; Supplementary Table S9). The proportional odds ordinal logistic regression likewise confirmed the association between PC1 and higher HM Total scores (OR = 1.87, 95% CI [1.25, 2.80], *p* = 0.002; Supplementary Table S10).

Additional sensitivity analyses addressing fasting status and glycaemic classification yielded PC1 estimates consistent in direction and magnitude with the primary analysis. Restriction to participants assessed while fasting (n = 62) yielded a similar point estimate, although with wider confidence intervals (B = +0.347, 95% CI [−0.050, +0.745], *p* = 0.086). When participants receiving glucose-lowering therapy were retained irrespective of fasting status, the results were similar (n = 64; B = +0.354, 95% CI [−0.033, +0.740], *p* = 0.072). After excluding participants receiving glucose-lowering therapy or with glucose ≥126 mg/dL (n = 84), the association between PC1 and HM Total score remained statistically significant (B = +0.339, 95% CI [+0.021, +0.656], *p* = 0.037; Supplementary Table S11).

Full results are reported in the Supplementary Materials.

### 3.4. Domain-Specific Associations: PC1 and Individual LC8+ Health Domains

To identify domain-specific associations between PC1 and individual LC8+ metrics, each of the eight binary HM scores was analysed using the primary fixed-effect specification, consisting of a generalised linear model with a binomial distribution and logit link, including PC1, age, sex, and recruitment site as fixed effects. To account for multiple testing across the eight domain-specific analyses, *p*-values for the PC1 coefficient were adjusted using the Benjamini–Hochberg false discovery rate procedure. Before correction for multiple testing, PC1 was positively associated with glycaemic health (OR = 2.47, 95% CI [1.32, 4.62], *p* = 0.005) and sleep health (OR = 1.99, 95% CI [1.05, 3.77], *p* = 0.035). After Benjamini–Hochberg correction across the eight LC8+ domains, only the association with glycaemic health remained statistically significant (adjusted *p* = 0.039), whereas the association with sleep health did not (adjusted *p* = 0.142). No significant associations were observed for the remaining LC8+ domains (Table 4).

In sensitivity analyses using generalised linear mixed-effects models with recruitment site as a random intercept, effect estimates were broadly similar in magnitude and direction to those obtained from the primary fixed-effects models. Although the association between PC1 and glycaemic health was nominally significant (OR = 2.29, 95% CI [1.25, 4.18], *p* = 0.007), it did not meet the Benjamini–Hochberg-adjusted significance threshold (adjusted *p* = 0.058). No other domain-level associations met the adjusted significance threshold (Supplementary Table S12).

Sensitivity analyses addressing fasting status and glycaemic classification showed directionally consistent associations. In participants assessed while fasting, the association between PC1 and meeting the glycaemic health criterion remained positive but was less precise (n = 62; OR = 1.75, 95% CI [0.86, 3.53], *p* = 0.121). When fasting participants were analysed while additionally retaining non-fasting participants receiving glucose-lowering therapy (n = 64), a similar estimate was observed (OR = 1.88, 95% CI [0.93, 3.80], *p* = 0.080). After excluding participants receiving glucose-lowering therapy or with glucose ≥126 mg/dL (n = 84), the association remained nominally significant (OR = 2.35, 95% CI [1.17, 4.68], *p* = 0.016; Supplementary Table S13).

An additional Firth bias-reduced logistic regression addressing sparse site-by-outcome cells yielded a similar PC1 effect estimate (Supplementary Table S14).

### 3.5. Determinants of Wearable-Derived PC1

To identify independent determinants of the wearable-derived PC1, a multivariable linear regression model with age, sex, BMI, and Godin–Shephard score was fitted. The model was statistically significant (F(4, 91) = 7.26, *p* < 0.001, R^2^ = 0.242, adjusted R^2^ = 0.209). Male sex (B = +0.662, 95% CI [+0.26, +1.07], *p* = 0.002) and higher Godin–Shephard score (B = +0.014, 95% CI [+0.01, +0.02], *p* = 0.002) were independently associated with higher PC1 values (Table 5). Neither age (*p* = 0.500) nor BMI (*p* = 0.548) was independently associated with PC1.

## 4. Discussion

In the present study, we identified three latent physiological dimensions from a single 24-h wearable device recording. Based on their loading patterns, these dimensions corresponded predominantly to HRV and nocturnal heart rate measures (PC1), sleep-related characteristics (PC2), and respiration-related indices (PC3), respectively. Among these, only the autonomic dimension represented by PC1 was associated with overall cardiovascular health as assessed by the LC8+ framework, independently of age and sex. Furthermore, PC1 showed a positive association with glycaemic health status. Collectively, these exploratory findings suggest that wearable-derived data provide complementary physiological information beyond traditional rhythm assessment and support the further investigation of wearable-derived physiological dimensions as candidate digital markers of cardiovascular health in preventive medicine.

Heart rate variability (HRV) is a well-established non-invasive marker of autonomic nervous system function and cardiovascular health [2]. Reduced HRV and elevated resting or nocturnal heart rate have been associated with cardiovascular disease [5], metabolic disorders [6], and increased mortality [20]. Rather than evaluating individual HRV indices separately, our study integrated multiple wearable-derived physiological variables into a single latent autonomic dimension. PC1, characterised by higher SDNN, higher RMSSD and lower nocturnal heart rate, was independently associated with the overall LC8+ score after adjustment for age and sex. These findings suggest that multiple wearable-derived physiological signals can be integrated into a single latent autonomic dimension associated with cardiovascular health. This approach is consistent with growing interest in integrating multidimensional wearable-derived physiological information to characterise cardiovascular physiology [3,21,22].

Importantly, the association between PC1 and cardiovascular health was independent of chronological age. Although ageing is typically accompanied by progressive reductions in vagal modulation and HRV, our findings indicate that the physiological information provided by PC1 extends beyond the expected effects of ageing, suggesting that the wearable-derived autonomic component reflects broader aspects of cardiovascular health rather than simply chronological ageing.

Among the individual LC8+ domains, PC1 showed the strongest association with glycaemic health status. The observed association between higher PC1 values and better glycaemic health is consistent with previous evidence linking autonomic regulation with glucose metabolism. Reduced HRV has consistently been associated with insulin resistance, impaired glucose regulation and incident type 2 diabetes in population-based cohorts [23,24], while a recent meta-analysis confirmed a substantial reduction in HRV across multiple autonomic domains in individuals with diabetes [25].

Although the association between PC1 and the LC8+ sleep domain did not remain statistically significant after correction for multiple testing, its direction is in keeping with previous evidence linking autonomic regulation to sleep health. Healthy sleep is characterised by nocturnal parasympathetic predominance and cardiovascular recovery, whereas sleep disruption and fragmentation are associated with sustained sympathetic activation and reduced heart rate variability [26]. Recent genetic evidence suggests that altered HRV may increase insomnia risk, supporting a link between autonomic dysfunction and impaired sleep regulation [27]. Further investigation in larger independent cohorts will be required to confirm the observed association between the wearable-derived autonomic component and sleep health.

To further characterise the physiological meaning of PC1, we explored its relationship with participant characteristics and lifestyle factors. Habitual physical activity, assessed using the Godin–Shephard Leisure-Time Physical Activity Questionnaire, was independently associated with higher PC1 scores after adjustment for age, sex and BMI. This observation is consistent with extensive evidence linking regular exercise to greater cardiac vagal modulation, higher HRV, and lowers resting heart rate through favourable autonomic adaptations [28,29], although the cross-sectional nature of the present study precludes inference regarding the direction or causality of this relationship.

This study has several limitations that should be considered when interpreting the findings. The cross-sectional design precludes causal inference; the observed associations between wearable ECG dimensions and LC8+ health outcomes may reflect reverse causation (e.g., better metabolic health enabling greater parasympathetic tone). Longitudinal studies are needed to determine whether wearable-derived physiological dimensions are associated with future glycaemic deterioration or incident cardiovascular events over time. Although the association between PC1 and the overall LC8+ score remained robust across multiple sensitivity analyses, the analyses of the individual LC8+ domains were exploratory and should be considered hypothesis-generating. Multiple statistical comparisons were performed across the individual LC8+ domains, and after adjustment for multiple testing, only the association with glycaemic health remained statistically significant in the primary fixed-effects analysis. However, the statistical significance of this association was sensitive to model specification: it did not remain significant after multiple-testing correction in the mixed-effects sensitivity analysis, despite an effect estimate of similar magnitude and direction. This specification dependence warrants caution in interpreting the robustness of the finding. Given the modest sample size and the multiplicity-adjusted selection of the glycaemic association, the observed odds ratio may overestimate the underlying effect size; accordingly, the confidence interval should be considered more informative than the point estimate itself. These findings therefore require confirmation in larger independent cohorts. Furthermore, the exploratory domain-specific analyses were restricted to PC1; therefore, the domain-specific correlates of PC2 and PC3 were not investigated. In addition, no statistical analysis plan was preregistered, and the exploratory findings should therefore be interpreted accordingly. The sample size (N = 97) was modest, and participation in wearable recording was voluntary, self-selected, and capacity-limited by device availability. Individuals who did not undergo wearable recording were not systematically tracked as eligible non-participants; consequently, a conventional participation rate could not be calculated, and selection bias cannot be excluded. Participants volunteering at preventive medicine events may also be more health-conscious and motivated than the broader community population. These features of recruitment may limit the generalisability of the findings and may have reduced variability in both wearable and clinical characteristics, potentially limiting the precision of the domain-specific analyses. The LC8+ health metrics are binary (pass/fail) thresholds applied to continuous biological variables. This dichotomisation reduces statistical power and may mask clinically meaningful graded associations. Moreover, several LC8+ domains incorporate treatment status or standardised clinical thresholds rather than raw physiological measurements alone. For example, glucose measurements were not uniformly obtained under fasting conditions, while blood pressure and cholesterol classifications also account for antihypertensive and lipid-lowering therapy. Consequently, analyses based on the underlying continuous measurements would not have been directly comparable across participants.

The latent physiological dimensions identified by PCA are data-driven constructs rather than validated physiological entities. Furthermore, the PCA-derived physiological dimensions were not directly evaluated against established autonomic indices, objective measures of cardiorespiratory fitness, or validated cardiovascular risk scores. Future studies should compare these approaches to determine the relative performance and potential incremental clinical value of the wearable-derived components. Moreover, the PCA was performed in a relatively small convenience sample with only modest sampling adequacy, as reflected by the KMO and individual MSA values. Although the resulting component structure was biologically interpretable and yielded effect estimates of similar magnitude and direction across the sensitivity analyses, it should be regarded as preliminary and requires replication and external validation in independent cohorts before broader generalisation. The mean SDNN values observed in this cohort were higher than those commonly reported in population-based studies. However, visual inspection of the data distribution did not identify extreme outliers, and sensitivity analyses excluding participants with markedly elevated SDNN values (>300 ms) yielded results comparable to the primary analyses. Participants were recruited from preventive medicine events and represented a relatively active population, as reflected by the high Godin–Shephard Leisure-Time Exercise Questionnaire scores. No restrictions regarding physical activity during the preceding days or on the day of ECG acquisition were imposed. Consequently, the habitual and recent physical activity in this cohort may have contributed to the observed HRV values. In addition, ECG recordings were processed using the manufacturer’s proprietary automated quality-control and artefact-correction pipeline. However, because the software does not report the number or proportion of corrected RR intervals, the extent of artefact editing could not be quantified at the individual recording level. In addition, some of the wearable-derived indices incorporated into the PCA (e.g., CVHRI and CEI) were proprietary outputs generated by the manufacturer’s algorithms. Although these indices have been evaluated for the detection of sleep-disordered breathing [16], their use as components of a broader latent physiological construct has not been independently validated. Although the regression models adjusted for age, sex, and recruitment site, residual confounding from unmeasured factors such as medication use (e.g., beta-blockers), cardiovascular comorbidities or sleep disorders cannot be excluded. In addition, the modest sample size did not permit robust age-stratified analyses, limiting conclusions regarding potential age-specific differences in the observed associations. Finally, the Godin–Shephard Leisure-Time Exercise Questionnaire, used in the exploratory secondary analysis, relies on self-report and is subject to recall and social-desirability bias; future studies should complement it with device-derived accelerometry measures.

## 5. Conclusions

Principal component analysis of eight wearable-derived physiological variables identified three latent physiological dimensions explaining 70.1% of the total variance. Among these, the autonomic dimension (PC1) was independently associated with overall cardiovascular health assessed by the LC8+ framework and showed the strongest association with glycaemic status. The independent association between PC1 and habitual physical activity, but not age, suggests a relationship between this wearable-derived physiological dimension and lifestyle behaviour, although the cross-sectional design precludes conclusions regarding directionality or causality. Collectively, these findings suggest that the multidimensional analysis of 24-h wearable recordings may provide complementary candidate digital markers of cardiometabolic health that warrant further evaluation in longitudinal and externally validated studies before their clinical application.

## Figures and Tables

**Table 1:** Participant characteristics.

Variable	Total
***Recruitment site***	
Gaeta	30 (30.9%)
Roma	25 (25.8%)
Ovindoli	22 (22.7%)
Roma Urbs Mundi	20 (20.6%)
***Demographics***	
Age (years)	51.3 ± 13.4
Sex, males	62 (63.9%)
BMI (kg/m^2^)	23.3 ± 3.0
Systolic BP (mmHg)	124.9 ± 15.5
Total cholesterol (mg/dL)	206.3 ± 46.2
Blood glucose (mg/dL)	102.9 ± 19.8
Fasting at glucose assessment	63 (64.9%)
Godin–Shephard score	39.5 ± 21.7
***LC8+ Health Metrics—n (%) meeting criterion***	
HM Smoking	69 (71.1%)
HM Diet	60 (61.9%)
HM Physical activity	82 (84.5%)
HM Blood pressure	48 (49.5%)
HM Cholesterol	31 (32.0%)
HM Glucose	71 (73.2%)
HM Sleep	78 (80.4%)
HM BMI	73 (75.3%)
HM Total score (0–8), mean ± SD	5.3 ± 1.5
***Wearable ECG variables***	
SDNN (ms)	209.9 ± 55.1
RMSSD (ms)	41.4 ± 15.9
Nocturnal HR (bpm)	59.6 ± 8.2
CVHRI (events/h)	14.9 ± 11.7
CEI (events/h)	5.1 ± 5.2
Sleep efficiency (%)	85.5 ± 7.6
WASO (min)	39.7 ± 40.9
Sleep duration (h)	6.4 ± 1.2

Data are presented as mean ± SD or n (%). Unless otherwise specified, data were available for all 97 participants. CVHRI, CEI, and WASO were available for 96 participants. BMI = body mass index; BP = blood pressure; LC8+ = Longevity Check-Up 8+; HM = health metric (0 = not meeting criterion, 1 = meeting criterion); SDNN = standard deviation of normal-to-normal intervals; RMSSD = root mean square of successive differences; HR = heart rate; CVHRI = Cyclic Variation of Heart Rate Index; CEI = Chest Effort Index; WASO = wake after sleep onset.

**Table 2:** Principal component loadings of the eight wearable-derived variables retained in the principal component analysis.

Variable	PC1	PC2	PC3
SDNN (ms)	0.9205 *	−0.0203	−0.2543
Nocturnal mean HR (bpm)	−0.8623 *	0.2368	0.0087
RMSSD (ms)	0.5468 *	0.1000	−0.5781 *
CVHRI (events/h)	0.4938	0.0789	0.6794 *
CEI (events/h)	0.2868	0.2902	0.6747 *
Sleep efficiency (%)	0.1082	−0.8233 *	0.1858
WASO (min)	−0.0637	0.8876 *	−0.0590
Mean sleep duration (h)	0.3747	0.5114 *	0.0129
Eigenvalue	2.3722	1.8841	1.3538
Variance explained (%)	29.65	23.55	16.92
Cumulative variance (%)	29.65	53.20	70.13

Loadings represent the correlation between each standardised variable and the retained principal component. PCA was performed on 96 participants with complete data for all eight wearable-derived variables. Parallel analysis supported retention of three components. Overall KMO = 0.4934; Bartlett’s test of sphericity: χ^2^(28) = 291.2705, *p* < 0.001. Variables with higher absolute loadings contribute more strongly to the interpretation of each component. * |Loadings| ≥ 0.50. CEI = Chest Effort Index; CVHRI = Cyclic Variation of Heart Rate Index; HR = heart rate; RMSSD = root mean square of successive RR interval differences; SDNN = standard deviation of normal-to-normal RR intervals; WASO = wake after sleep onset.

**Table 3:** Linear regression model examining the association between wearable-derived principal components and overall HM Total score.

Fixed Effect	B (SE)	95% CI	*p*
PC1	+0.402 (0.150)	[+0.103, +0.700]	0.009 **
PC2	−0.162 (0.149)	[−0.458, +0.133]	0.278
PC3	−0.027 (0.172)	[−0.369, +0.314]	0.875
Age (years)	−0.026 (0.012)	[−0.050, −0.002]	0.037 *
Sex (male vs. female)	−0.668 (0.325)	[−1.314, −0.021]	0.043 *
Ovindoli vs. Gaeta (ref)	+0.789 (0.401)	[−0.009, +1.586]	0.053
Roma vs. Gaeta (ref)	+0.370 (0.395)	[−0.414, +1.154]	0.351
Roma Urbs vs. Gaeta (ref)	−0.420 (0.404)	[−1.222, +0.383]	0.301

Model fit: N = 96; R^2^ = 0.226; adjusted R^2^ = 0.155; F(8,87) = 3.175; *p* = 0.003. B = unstandardised regression coefficient; SE = standard error; CI = confidence interval. HM Total score range 0–8 (higher = better cardiometabolic health). * *p* < 0.05; ** *p* < 0.01.

**Table 4:** Primary fixed-effect logistic regression analyses examining the association between PC1 and individual Longevity Check-Up 8+ (LC8+) health domains.

LC8+ Domain	OR (PC1)	95% CI	*p*	BH-Adjusted *p*
HM Glucose	2.47	[1.32, 4.62]	0.005 **	0.039 *
HM Sleep	1.99	[1.05, 3.77]	0.035 *	0.142
HM Physical activity	1.74	[0.93, 3.26]	0.083	0.222
HM BMI	1.38	[0.81, 2.36]	0.234	0.468
HM Diet	1.24	[0.78, 1.98]	0.357	0.571
HM Blood pressure	1.00	[0.61, 1.63]	0.993	0.993
HM Smoking	0.95	[0.55, 1.62]	0.841	0.993
HM Cholesterol	0.96	[0.57, 1.62]	0.887	0.993

N = 96. Models included PC1, age, sex, and recruitment site as fixed effects. Results are presented as odds ratios (ORs) with 95% confidence intervals (CIs), Wald *p*-values, and Benjamini–Hochberg (BH) false discovery rate-adjusted *p*-values. BH correction was applied across the eight domain-specific analyses. * *p* < 0.05; ** *p* < 0.01. PC1 = principal component 1. HM = health metric (binary 0/1).

**Table 5:** Multiple linear regression: demographic and lifestyle predictors of wearable-derived PC1.

Predictor	B (SE)	95% CI	*p*
Age (years)	−0.005 (0.007)	[−0.018, 0.009]	0.500
Sex (male)	+0.662 (0.203)	[0.258, 1.066]	0.002
BMI (kg/m^2^)	−0.020 (0.033)	[−0.085, 0.045]	0.548
Godin–Shephard score	+0.014 (0.004)	[0.005, 0.023]	0.002

N = 96; R^2^ = 0.242; adjusted R^2^ = 0.209; F(4, 91) = 7.26, *p* < 0.001. B = unstandardised coefficient; SE = standard error; BMI = body mass index; CI = confidence interval.

## Data Availability

The dataset supporting the conclusions of this article is available from the corresponding author upon reasonable request.

## Supplementary Materials

The following supporting information can be downloaded at: https://www.mdpi.com/article/10.3390/medsci14050540/s1, Supplementary Table S1: Definition and assessment of the eight health metric (HM) domains included in the Longevity Check-Up 8+ (LC8+) cardiovascular health framework. Supplementary Table S2: Wearable-derived variables included in the principal component analysis. Supplementary Table S3. Participant characteristics stratified by recruitment site. Sensitivity Analyses: PCA models. Supplementary Table S4. Sampling adequacy and principal component loadings for the primary PCA model. PCA after excluding CEI. Supplementary Table S5. Sampling adequacy and principal component loadings for the PCA model after the exclusion of the Chest Effort Index (CEI). PCA after excluding CEI and RMSSD. Supplementary Table S6. Sampling adequacy and principal component loadings for the PCA model after the exclusion of the Chest Effort Index (CEI) and RMSSD. PCA after excluding participants with SDNN > 300 ms. Supplementary Table S7. Sampling adequacy and principal component loadings for the PCA model after the exclusion of participants with SDNN > 300 ms. Linear regression model: residual diagnostics and sensitivity analyses. Figure S1. Q–Q plot of standardised residuals from the primary linear model. Supplementary Table S8. Linear mixed model: associations between wearable-derived principal components and overall LC8+ health score (HM Total). Supplementary Table S9. Multiple linear regression model after the exclusion of participants with SDNN > 300 ms. Supplementary Table S10. Sensitivity analysis using proportional odds ordinal logistic regression with HM Total as the outcome. Sensitivity analyses addressing fasting status and glycaemic classification. Supplementary Table S11. Sensitivity analyses of the association between wearable-derived principal components and overall HM Total score according to fasting status and glycaemic classification. Domain-Specific Associations of PC1: Sensitivity analyses. Supplementary Table S12. Sensitivity analyses of the associations between PC1 and individual Longevity Check-Up 8+ (LC8+) health domains using generalised linear mixed-effects models with recruitment site included as a random intercept. Supplementary Table S13. Glycaemic-domain logistic sensitivity analyses. Firth penalised logistic regression sensitivity analysis. Supplementary Table S14. Sensitivity analysis of the association between PC1 and HM Glucose using Firth bias-reduced logistic regression.

## Abbreviations

The following abbreviations are used in this manuscript:

BMI	Body Mass Index
CEI	Chest Effort Index
CI	Confidence Interval
CVHRI	Cyclic Variation of Heart Rate Index
ECG	Electrocardiography
GLMM	Generalised Linear Mixed Models
HM	Health Metric
HR	Heart Rate
HRV	Heart Rate Variability
KMO	Kaiser–Meyer–Olkin
LC8+	Longevity Check-Up 8+
LE8	Life’s Essential 8
LMM	Linear Mixed Model
OR	Odds Ratio
PCA	Principal Component Analysis
RMSSD	Root Mean Square of Successive RR interval Differences
SD	Standard Deviation
SDNN	Standard Deviation of all Normal-to-Normal RR intervals over 24 h
WASO	Wake After Sleep Onset
