# Supplementary material for: A Wearable-Derived Autonomic Component Is Associated with Cardiovascular Health in Community-Dwelling Adults: A Cross-Sectional Analysis from the Longevity Check-Up 8+ Study
Source: Med Sci (Basel). 2026 Sep 2;14(5):540. doi: 10.3390/medsci14050540 (PMC13609875; doi:10.3390/medsci14050540)
Supplement: Supplementary file 1 [file medsci-14-00540-s001.zip › medsci-4453323-supplementary.pdf]

Supplementary Materials

Supplementary Table S1. Definition and assessment of the eight health metric (HM) domains included in the Longevity Check-Up 8+ (LC8+) cardiovascular health framework.

Health metric	Assessment method	Criterion for meeting target (1 point)
**Smoking**	Self-reported smoking status collected through a standardized questionnaire	Never smoker or former smoker; current smokers scored 0.
**Diet**	Brief dietary questionnaire assessing daily fruit and vegetable consumption	Consumption of ≥3 daily servings of fruit and/or vegetables.
**Physical activity**	Standardized questionnaire on habitual exercise	Regular physical activity performed ≥2 times/week (≥30 min/session).
**Body mass index (BMI)**	Weight and height measured on site using calibrated equipment; BMI calculated as kg/m^2	BMI <25 kg/m^2 (with age-specific threshold according to the LC8+ protocol, when applicable).
**Blood pressure**	Seated blood pressure measured using an automated sphygmomanometer; antihypertensive therapy recorded	Untreated blood pressure <120/80 mmHg and no antihypertensive treatment.
**Blood cholesterol**	Capillary total cholesterol measured using a point-of-care device; lipid-lowering therapy recorded	Total cholesterol <200 mg/dL and no lipid-lowering therapy.
**Blood glucose**	Capillary glucose measurement (fasting status recorded); glucose-lowering therapy recorded	Blood glucose <100 mg/dL (fasting) or <200 mg/dL (non-fasting), without glucose-lowering therapy.
**Sleep quality**	Single self-reported question referring to overall sleep quality during the previous month	Self-reported sleep quality rated as very good or fairly good.

Supplementary Table S2. Wearable-derived physiological variables included in the principal component analysis.

Physiological category	Variable	Unit	Description
**Autonomic function**	Mean nocturnal HR	bpm	Mean heart rate recorded during the nocturnal sleep period.
	SDNN	ms	Standard deviation of all normal-to-normal (NN) RR intervals over the entire 24-h wearable recording.
	RMSSD	ms	Root mean square of successive differences between adjacent normal RR intervals over the entire 24-h wearable recording
**Sleep parameters**	Sleep efficiency	%	Percentage of total time in bed spent asleep, calculated as total sleep time divided by time in bed × 100.
	WASO	min	Total time spent awake after sleep onset until final awakening.
	Mean sleep duration	h	Total sleep time during the monitored night.
**Nocturnal respiratory parameters**	CVHRI	events/h	Mean number of cyclic variation of heart rate (CVHR) events detected during sleep per hour of sleep using the Rooti Rx algorithm.
	CEI	events/h	Mean number of chest effort events detected from thoracic motion signals acquired by the three-axis accelerometer during sleep, expressed per hour of sleep.

CVHRI and CEI are proprietary indices automatically derived by the Rooti Rx analysis algorithm for estimating nocturnal cardiorespiratory events and respiratory effort, respectively [1]. Abbreviations: CEI, Chest Effort Index; CVHRI, Cyclic Variation of Heart Rate Index; HR, heart rate; NN, normal-to-normal; RMSSD, root mean square of successive RR interval differences; SDNN, standard deviation of normal-to-normal RR intervals; WASO, wake after sleep onset.

Supplementary Table S3. Participant characteristics stratified by recruitment site (N = 97).

Variable	Gaeta (N = 30)	Roma (N = 25)	Ovindoli (N = 22)	Roma Urbs Mundi (N = 20)
**Demographics**				
Age (years)	52.4 ± 9.8	50.0 ± 18.1	55.0 ± 12.9	47.1 ± 11.3
Sex, males	24 (80.0%)	9 (36.0%)	15 (68.2%)	14 (70.0%)
BMI (kg/m²)	23.4 ± 2.3	22.8 ± 2.9	23.0 ± 3.1	23.9 ± 4.2
**Clinical**				
Systolic BP (mmHg)	125.0 ± 14.6	118.0 ± 9.8	123.4 ± 17.3	135.0 ± 16.4
Total cholesterol (mg/dL)	205.3 ± 41.8	214.9 ± 47.6	195.0 ± 41.2	209.5 ± 56.0
Blood glucose (mg/dL)	105.4 ± 20.6	103.9 ± 25.1	103.7 ± 16.1	96.9 ± 14.1
Fasting at glucose assessment	16 (53.3%)	19 (76.0%)	13 (59.1%)	15 (75.0%)
Godin–Shephard score	44.9 ± 25.5	34.8 ± 12.8	29.7 ± 8.8	47.8 ± 28.8
**LC8+ Health Metrics — n (%) meeting criterion**				
HM Smoking	22 (73.3%)	22 (88.0%)	18 (81.8%)	7 (35.0%)
HM Diet	18 (60.0%)	16 (64.0%)	15 (68.2%)	11 (55.0%)
HM Physical activity	24 (80.0%)	24 (96.0%)	19 (86.4%)	15 (75.0%)
HM Blood pressure	14 (46.7%)	17 (68.0%)	13 (59.1%)	4 (20.0%)
HM Cholesterol	9 (30.0%)	5 (20.0%)	9 (40.9%)	8 (40.0%)
HM Glucose	22 (73.3%)	17 (68.0%)	14 (63.6%)	18 (90.0%)
HM Sleep	20 (66.7%)	20 (80.0%)	19 (86.4%)	19 (95.0%)
HM BMI	23 (76.7%)	19 (76.0%)	18 (81.8%)	13 (65.0%)
HM Total (0–8)	5.1 ± 1.6	5.6 ± 1.4	5.7 ± 1.5	4.8 ± 1.2
**Wearable ECG variables**				
SDNN (ms)	229.0 ± 55.9	191.8 ± 40.7	202.5 ± 62.6	211.9 ± 55.6
RMSSD (ms)	39.7 ± 14.3	44.1 ± 12.0	39.4 ± 16.6	42.8 ± 21.4
Nocturnal HR (bpm)	58.1 ± 9.1	61.3 ± 5.9	60.6 ± 8.7	58.6 ± 8.7
CVHRI (events/h)	15.7 ± 11.3	10.4 ± 7.9	19.4 ± 15.9	14.5 ± 9.0
CEI (events/h)	5.5 ± 4.8	3.9 ± 1.8	7.9 ± 8.4	2.8 ± 1.5
Sleep efficiency (%)	87.1 ± 6.2	86.6 ± 4.9	82.7 ± 11.9	84.7 ± 5.4
WASO (min)	36.0 ± 27.6	35.0 ± 14.6	54.9 ± 74.4	33.9 ± 22.0
Sleep duration (h)	6.5 ± 1.0	5.9 ± 0.9	7.2 ± 1.6	5.8 ± 0.8

Data are presented as mean ± SD or n (%), as appropriate. CVHRI, CEI, and WASO were unavailable for one participant at Roma Urbs Mundi. BMI = body mass index; BP = blood pressure; SDNN = standard deviation of normal-to-normal intervals; RMSSD = root mean square of successive differences; HR = heart rate; CVHRI = Cyclic Variation of Heart Rate Index; CEI = Chest Effort Index; WASO = wake after sleep onset; HM = health metric (binary 0/1).

Sensitivity Analyses: PCA models.

In the primary PCA model, based on 96 participants with complete data for all eight wearable-derived variables, the overall KMO measure of sampling adequacy was 0.493, and Bartlett's test of sphericity was significant ($p < 0.001$). Parallel analysis supported the retention of three principal components, which collectively explained 70.1% of the total variance. Individual measures of sampling adequacy (MSA) are reported in Supplementary Table S4. CEI (MSA = 0.408) and RMSSD (MSA = 0.430) showed the lowest sampling adequacy.

Supplementary Table S4. Sampling adequacy and principal component loadings for the primary PCA model.

Variable	PC1	PC2	PC3	MSA
SDNN (ms)	+0.9205*	−0.0203	−0.2543	0.4745
Nocturnal mean HR (bpm)	−0.8623*	+0.2368	+0.0087	0.4931
RMSSD (ms)	+0.5468*	+0.1000	−0.5781*	0.4305
CVHRI (events/h)	+0.4938	+0.0789	+0.6794*	0.6295
CEI (events/h)	+0.2868	+0.2902	+0.6747*	0.4078
Sleep efficiency (%)	+0.1082	−0.8233*	+0.1858	0.5351
WASO (min)	−0.0637	+0.8876*	−0.0590	0.5103
Mean sleep duration (h)	+0.3747	+0.5114*	+0.0129	0.5156
Eigenvalue	2.3722	1.8841	1.3538	—
Variance explained (%)	29.65	23.55	16.92	—
Cumulative variance (%)	29.65	53.20	70.13	—

Analytic sample: n = 96 complete cases. Overall KMO = 0.4934. Bartlett’s test: $\chi^2(28) = 291.2705$, $p < 0.001$. *|loading| ≥ 0.50. CEI = Chest Effort Index; CVHRI = Cyclic Variation of Heart Rate Index; HR = heart rate; MSA = measure of sampling adequacy; PC = principal component; RMSSD = root mean square of successive RR interval differences; SDNN = standard deviation of normal-to-normal RR intervals; WASO = wake after sleep onset.

Sensitivity analyses were performed by sequentially excluding these variables. Although exclusion of CEI alone and CEI plus RMSSD increased the overall KMO above the conventional threshold of 0.500, the overall component structure changed. Nevertheless, the variables defining PC1, particularly SDNN and mean nocturnal heart rate, retained similar loadings across all analyses (Supplementary Tables S5–S6).

PCA after excluding CEI

Principal component analysis was repeated after excluding CEI, the variable with the lowest measure of sampling adequacy (MSA) in the primary analysis. Parallel analysis supported the retention of two components, which together explained 59.6% of the total variance. Overall sampling adequacy improved (KMO = 0.514), while Bartlett's test of sphericity remained significant ($p < 0.001$).

Supplementary Table S5. Sampling adequacy and principal component loadings for the PCA model after the exclusion of the Chest Effort Index (CEI).

Variable	PC1	PC2	MSA
SDNN (ms)	0.9440*	0.1029	0.5033
Nocturnal mean HR (bpm)	−0.8837*	0.1561	0.5063
RMSSD (ms)	0.5701*	0.2177	0.4592
CVHRI (events/h)	0.4316	0.0154	0.6979
Sleep efficiency (%)	0.1635	−0.8382*	0.5340
WASO (min)	−0.1336	0.8873*	0.5018
Mean sleep duration (h)	0.3175	0.5212*	0.5696
Eigenvalue	2.3286	1.8441	—
Variance explained (%)	33.27	26.34	—
Cumulative variance (%)	33.27	59.61	—

Analytic sample: n = 96 complete cases. Overall KMO = 0.5143. Bartlett's test: $\chi^2(21)$ = 266.7282, $p < 0.001$. *|loading| ≥ 0.50. CEI = Chest Effort Index; CVHRI = Cyclic Variation of Heart Rate Index; HR = heart rate; MSA = measure of sampling adequacy; PC = principal component; RMSSD = root mean square of successive RR interval differences; SDNN = standard deviation of normal-to-normal RR intervals; WASO = wake after sleep onset.

PCA after excluding CEI and RMSSD

Further exclusion of RMSSD, the variable with the second-lowest MSA in the primary analysis, resulted in a modest additional increase in the overall KMO (0.515). Bartlett's test of sphericity remained significant ($p < 0.001$). Parallel analysis supported the retention of two components, which together explained 65.7% of the total variance. Although the overall component structure changed, the variables defining PC1, particularly SDNN and mean nocturnal heart rate, retained similar loadings across these analyses (Supplementary Tables S5–S6).

Supplementary Table S6. Sampling adequacy and principal component loadings for the PCA model after the exclusion of the Chest Effort Index (CEI) and RMSSD.

Variable	PC1	PC2	MSA
SDNN (ms)	0.8695*	0.2845	0.4992
Nocturnal mean HR (bpm)	−0.9239*	−0.0652	0.5053
CVHRI (events/h)	0.5299*	0.2117	0.6548
Sleep efficiency (%)	0.3461	−0.7805*	0.5332
WASO (min)	−0.3014	0.8575*	0.5221
Mean sleep duration (h)	0.1897	0.5730*	0.4459
Eigenvalue	2.1371	1.8028	—
Variance explained (%)	35.62	30.05	—
Cumulative variance (%)	35.62	65.66	—

Analytic sample: n = 96 complete cases. Overall KMO = 0.5150. Bartlett's test: $\chi^2(15)$ = 216.6763, $p < 0.001$. *|loading| ≥ 0.50. CEI = Chest Effort Index; CVHRI = Cyclic Variation of Heart Rate Index; HR = heart rate; MSA = measure of sampling adequacy; PC = principal component; RMSSD = root mean square of successive RR interval differences; SDNN = standard deviation of normal-to-normal RR intervals; WASO = wake after sleep onset.

PCA after excluding participants with SDNN>300ms

In addition, the PCA was repeated after excluding seven participants with SDNN values >300 ms, resulting in an analytic sample of 89 participants. Parallel analysis again supported the retention of three components, which collectively explained 69.2% of the total variance. The overall KMO was 0.468, and Bartlett's test of sphericity remained significant ($p < 0.001$). The overall pattern of component loadings remained broadly comparable to that of the primary PCA, particularly for PC1 (Supplementary Table S7).

Supplementary Table S7. Sampling adequacy and principal component loadings for the PCA model after the exclusion of participants with SDNN>300ms.

Variable	PC1	PC2	PC3	MSA
SDNN (ms)	0.9109*	−0.1329	−0.2365	0.4413
Nocturnal mean HR (bpm)	−0.8220*	0.3359	−0.0757	0.4523
RMSSD (ms)	0.4961	0.0092	−0.5954*	0.3805
CVHRI (events/h)	0.4079	0.1945	0.7211*	0.5890
CEI (events/h)	0.2906	0.3448	0.6169*	0.3882
Sleep efficiency (%)	−0.0017	−0.8074*	0.2445	0.5412
WASO (min)	0.0008	0.8778*	−0.1485	0.5314
Mean sleep duration (h)	0.4462	0.4729	−0.0545	0.4757
Eigenvalue	2.2015	1.9334	1.4015	—
Variance explained (%)	27.52	24.17	17.52	—
Cumulative variance (%)	27.52	51.69	69.20	—

Analytic sample: n = 89 (7 participants excluded from the 96-person primary PCA sample). Overall KMO = 0.4682. Bartlett's test: $\chi^2(28) = 248.5586$, $p < 0.001$. *|loading| ≥ 0.50. CEI = Chest Effort Index; CVHRI = Cyclic Variation of Heart Rate Index; HR = heart rate; MSA = measure of sampling adequacy; PC = principal component; RMSSD = root mean square of successive RR interval differences; SDNN = standard deviation of normal-to-normal RR intervals; WASO = wake after sleep onset.

Linear regression: residual diagnostics and sensitivity analyses

Primary model residual diagnostics confirmed that the assumptions of the linear regression model were adequately met: residuals showed no evidence of departure from normality (Shapiro–Wilk W = 0.994, p = 0.930), no evidence of heteroscedasticity was observed (Breusch–Pagan BP = 10.184, df = 8, p = 0.252), and multicollinearity was negligible (maximum adjusted GVIF = 1.244). No highly influential observations were identified (maximum Cook's distance = 0.086). QQ plot of standardised residuals is provided in Supplementary Figure S1.

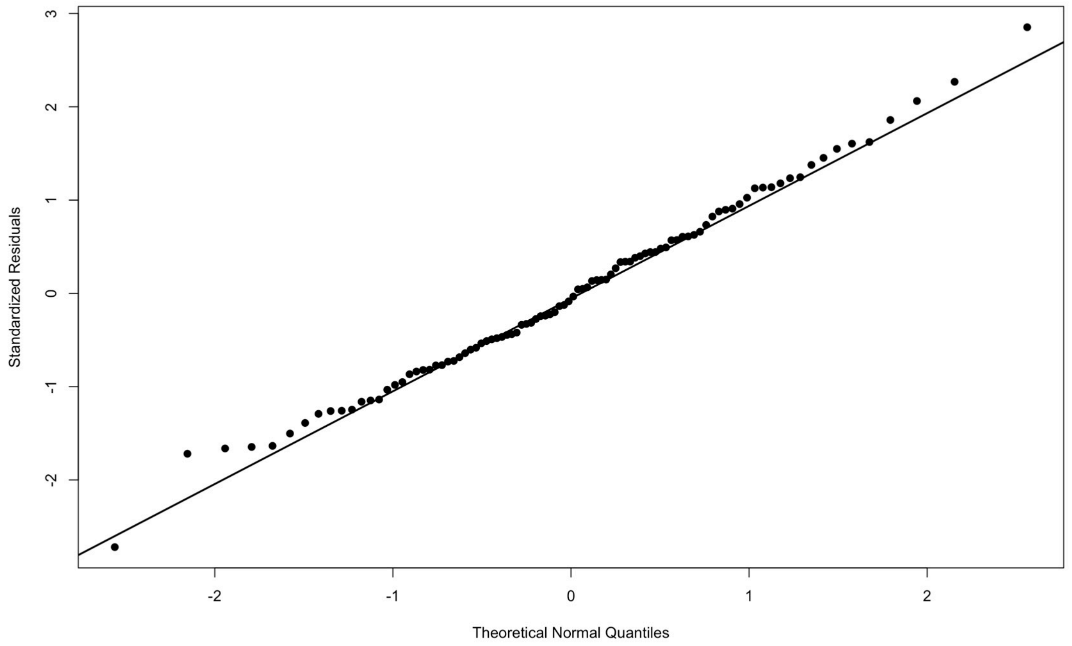

Figure S1. Q-Q plot of standardised residuals from the primary linear model. Standardised residuals plotted against theoretical normal quantiles for the primary linear model (HM Total ~ PC1 + PC2 + PC3 + age + sex + recruitment site). Points closely follow the diagonal reference line, indicating that the normality assumption is satisfied (Shapiro-Wilk W = 0.994, p = 0.930).

To evaluate the robustness of the primary findings, multiple sensitivity analyses were performed. First, the primary linear regression model was re-estimated using a linear mixed-effects model (LMM) including recruitment site as a random intercept, to account for potential clustering of participants within recruitment sites. Second, the primary linear regression model was repeated after excluding participants with SDNN values >300 ms to evaluate the potential influence of extreme HRV values. Third, HM Total was analysed as an ordinal outcome using proportional odds ordinal logistic regression to evaluate whether the association with PC1 was consistent when the ordered nature of the score was considered.

The linear mixed-effects model with recruitment site modelled as a random intercept confirmed that PC1 remained independently associated with overall HM Total (B = +0.408, 95% CI [+0.114, +0.702], p = 0.008; Supplementary Table S8), after adjustment for PC2, PC3, age, and sex. PC2 (B = −0.118, p = 0.417) and PC3 (B = +0.008, p = 0.961) were not independently associated with HM Total.

Age (B = −0.025, p = 0.044) and male sex (B = −0.712, p = 0.029) were also significant fixed effects. The estimated recruitment-site ICC was 0.078.

Supplementary Table S8. Linear mixed model: associations between wearable-derived principal components and overall Longevity Check-Up 8+ health score (HM Total).

Fixed effect	B (SE)	95% CI	p
PC1	+0.408 (0.150)	[+0.114, +0.702]	0.008**
PC2	−0.118 (0.145)	[−0.401, +0.166]	0.417
PC3	+0.008 (0.170)	[−0.324, +0.341]	0.961
Age (years)	−0.025 (0.012)	[−0.049, −0.001]	0.044*
Sex (male vs. female)	−0.712 (0.321)	[−1.340, −0.084]	0.029*

Analytic sample: n = 96 complete cases. Linear mixed-effects model fitted by restricted maximum likelihood (REML), with recruitment site modelled as a random intercept. The model included PC1, PC2, PC3, age, and sex as fixed effects. ICC = 0.078; marginal R^2 = 0.151; conditional R^2 = 0.217. HM (Health Metric) Total range: 0–8 (higher scores indicate better cardiometabolic health). B = unstandardised regression coefficient; SE = standard error; CI = confidence interval; PC = principal component. *p < 0.05; **p < 0.01.

The linear regression model repeated after excluding participants with SDNN values >300 ms (7 participants excluded; n = 89) confirmed that PC1 remained independently associated with HM Total (B = +0.410, 95% CI [+0.052, +0.767], p = 0.025), whereas PC2 and PC3 remained non-significant. Model assumptions continued to be adequately satisfied, with no evidence of departure from normality or heteroscedasticity, negligible multicollinearity, and no highly influential observations.

Supplementary Table S9. Multiple linear regression model after exclusion of participants with SDNN >300 ms.

Fixed effect	B (SE)	95% CI	p
PC1	+0.410 (0.180)	[+0.052, +0.767]	0.025*
PC2	−0.142 (0.155)	[−0.450, +0.165]	0.360
PC3	−0.067 (0.187)	[−0.439, +0.305]	0.720
Age (years)	−0.027 (0.013)	[−0.052, −0.001]	0.042*
Sex (male vs. female)	−0.661 (0.343)	[−1.343, +0.021]	0.057
Group			
Ovindoli vs. Gaeta (ref)	+0.733 (0.440)	[−0.141, +1.608]	0.099
Roma vs. Gaeta (ref)	+0.360 (0.415)	[−0.467, +1.187]	0.389
Roma Urbs vs. Gaeta (ref)	−0.319 (0.436)	[−1.187, +0.549]	0.466

Model fit: N = 89; R^2 = 0.214; adjusted R^2 = 0.135; $F(8,80)$ = 2.717, p = 0.011. Residual diagnostics supported the adequacy of the model, with no evidence of departure from normality (Shapiro–Wilk W = 0.991, p = 0.807), no evidence of heteroscedasticity (Breusch–Pagan BP = 10.236, df = 8, p = 0.249), negligible multicollinearity (maximum adjusted GVIF = 1.220), and no highly influential observations (maximum Cook's distance = 0.092). HM Total range: 0–8 (higher scores indicate better cardiometabolic health). B = unstandardised regression coefficient; SE = standard error; CI = confidence interval. *p < 0.05.

The ordinal logistic regression yielded findings consistent with the primary linear regression analysis (Supplementary Table S10). PC1 remained independently associated with higher HM Total scores (OR = 1.87, 95% CI [1.25, 2.80], $p = 0.002$), whereas PC2 and PC3 were not significantly associated with the outcome (Supplementary Table S10). These results support the robustness of the observed association between the autonomic component represented by PC1 and overall cardiometabolic health across different modelling approaches.

Supplementary Table S10. Sensitivity analysis using proportional odds ordinal logistic regression with HM Total as the outcome.

Fixed effect	OR	95% CI	p
PC1	1.87	[1.25, 2.80]	0.002**
PC2	0.84	[0.56, 1.24]	0.376
PC3	0.86	[0.56, 1.33]	0.500
Age (years)	0.97	[0.94, 1.01]	0.099
Sex (male vs. female)	0.36	[0.15, 0.86]	0.021*
**Group**			
Ovindoli vs. Gaeta (ref)	3.35	[1.14, 9.79]	0.027*
Roma vs. Gaeta (ref)	1.99	[0.71, 5.59]	0.189
Roma Urbs vs. Gaeta (ref)	0.66	[0.23, 1.90]	0.445

Model fit: N = 96; likelihood-ratio $\chi^2(8) = 25.35$, $p = 0.001$; McFadden's pseudo-$R^2 = 0.076$; AIC = 334.51; BIC = 370.41. Proportional odds ordinal logistic regression with HM (Health metric) Total treated as an ordered outcome and adjusted for PC1, PC2, PC3, age, sex, and recruitment site. Odds ratios >1 indicate higher odds of being in a higher HM Total category. OR = odds ratio; CI = confidence interval; PC = principal component. *$p < 0.05$; **$p < 0.01$.

Sensitivity analyses addressing fasting status and glycaemic classification

Among the 96 participants included in the PCA-based analyses, 62 (64.6%) were assessed while fasting and 34 (35.4%) while non-fasting. Four participants were receiving glucose-lowering therapy and nine had glucose ≥126 mg/dL; 12 participants met at least one of these latter two criteria. To assess the robustness of the primary association to fasting status and glycaemic classification, the primary linear regression model was repeated under three alternative sample definitions. First, the analysis was restricted to participants assessed while fasting (n = 62). Second, the fasting-restricted analysis was repeated while retaining participants receiving glucose-lowering therapy, because their HM Glucose classification was determined by treatment status irrespective of fasting status (n = 64). Third, participants receiving glucose-lowering therapy or with measured glucose ≥126 mg/dL were excluded (n = 84). All models retained the same specification as the primary analysis, including PC1, PC2, PC3, age, sex, and recruitment site as fixed effects (Supplementary Table S11).

Supplementary Table S11. Sensitivity analyses of the association between wearable-derived principal components and overall HM Total score according to fasting status and glycaemic classification.

Analysis	N	Component	B (SE)	95% CI	p
Fasting participants only	62	PC1	+0.347 (0.198)	[−0.050, +0.745]	0.086
		PC2	+0.032 (0.190)	[−0.348, +0.413]	0.866
		PC3	−0.131 (0.193)	[−0.517, +0.256]	0.500
Fasting participants plus participants receiving glucose-lowering therapy	64	PC1	+0.354 (0.193)	[−0.033, +0.740]	0.072
		PC2	+0.029 (0.186)	[−0.344, +0.403]	0.875
		PC3	−0.140 (0.187)	[−0.515, +0.236]	0.459
Excluding glucose-lowering therapy or glucose ≥126 mg/dL	84	PC1	+0.339 (0.159)	[+0.021, +0.656]	0.037*
		PC2	−0.141 (0.146)	[−0.432, +0.150]	0.338
		PC3	−0.105 (0.176)	[−0.455, +0.245]	0.551

Values are unstandardised regression coefficients (B) with standard errors (SE) and 95% confidence intervals (CI). All models included PC1, PC2, PC3, age, sex, and recruitment site as fixed effects. HM = Health Metric; PC = principal component. In the second analysis, participants receiving glucose-lowering therapy were retained irrespective of fasting status because treatment status determined their HM Glucose classification independently of the measured glucose value. The third analysis excluded participants receiving glucose-lowering therapy or with measured glucose ≥126 mg/dL.

Domain-Specific Associations of PC1: sensitivity analyses

The eight domain-specific analyses were repeated using generalised linear mixed-effects models including recruitment site as a random intercept. The magnitude and direction of the estimated effects were broadly consistent with those observed in the primary fixed-effects analyses. Glycaemic health showed the strongest association with PC1 (OR = 2.29, 95% CI [1.25–4.18], p = 0.007), while the estimate for sleep health was also positive (OR = 1.85, 95% CI [1.00–3.40], p = 0.049). However, after Benjamini–Hochberg correction across the eight mixed-effects models, none of the associations remained statistically significant. All models were successfully fitted; boundary (singular) fits were observed in four of the eight models, likely reflecting the limited number of recruitment sites (n = 4) (Supplementary Table S12).

Supplementary Table S12. Sensitivity analyses of the associations between PC1 and individual Longevity Check-Up 8+ (LC8+) health domains using generalised linear mixed-effects models with recruitment site included as a random intercept.

Domain	OR	95% CI	p	BH-adjusted p	Singular fit
HM Glucose	2.29	[1.25, 4.18]	0.007	0.058	No
HM Sleep	1.85	[1.00, 3.40]	0.049	0.197	No
HM Physical activity	1.70	[0.90, 3.18]	0.099	0.265	Yes
HM BMI	1.43	[0.86, 2.39]	0.173	0.345	Yes
HM Diet	1.25	[0.79, 1.98]	0.330	0.528	Yes
HM Blood pressure	1.01	[0.62, 1.63]	0.980	0.980	No

HM Smoking	0.95	[0.57, 1.61]	0.860	0.980	No
HM Cholesterol	1.02	[0.61, 1.71]	0.928	0.980	Yes

N = 96. Models included PC1, age, and sex as fixed effects, with recruitment site included as a random intercept. Results are presented as odds ratios (ORs) with 95% confidence intervals (CIs), Wald p-values, and Benjamini–Hochberg (BH) false discovery rate-adjusted p-values for the PC1 coefficient. BH correction was applied across the eight domain-specific mixed-effects models. The singular-fit column indicates models with a boundary (singular) fit, corresponding to an estimated recruitment-site random-intercept variance of zero and likely reflecting the limited number of recruitment sites (n = 4). PC1 = Principal Component 1; HM = health metric.

The glycaemic-domain logistic regression was additionally repeated under alternative sample definitions addressing fasting status and glycaemic classification. The analysis was first restricted to participants assessed while fasting (n = 62). A second analysis retained the fasting participants together with the two non-fasting participants receiving glucose-lowering therapy (n = 64), whose HM Glucose classification was determined by treatment status irrespective of fasting status. Finally, participants receiving glucose-lowering therapy or with glucose ≥126 mg/dL were excluded (n = 84). All models retained the same specification as the primary domain-specific logistic regression (Supplementary Table S13).

Supplementary Table S13. Glycaemic-domain logistic sensitivity analyses.

**Analysis**	**N**	**PC1 OR**	**95% CI**	**p (PC1)**	**McFadden R^2**	**LR $\chi^2(6)$**	**p (model)**
Primary full sample	96	2.47	[1.32, 4.62]	0.005	0.160	17.587	0.007
Fasting participants only	62	1.75	[0.86, 3.53]	0.121	0.151	12.319	0.055
Fasting participants plus non-fasting participants receiving glucose-lowering therapy	64	1.88	[0.93, 3.80]	0.080	0.166	14.212	0.027
Excluding participants receiving glucose-lowering therapy or with glucose ≥126 mg/dL	84	2.35	[1.17, 4.68]	0.016	0.151	13.520	0.035

OR = odds ratio; CI = confidence interval; LR = likelihood-ratio test. Models included PC1, age, sex, and recruitment site. ORs refer to the odds of meeting the HM Glucose criterion per 1-SD increase in PC1. Reported p values are nominal.

Firth penalized logistic regression sensitivity analysis

Because of sparse site-by-outcome cells in the domain-specific analyses, an additional sensitivity analysis was performed for the association between PC1 and the HM Glucose criterion using Firth bias-reduced penalized logistic regression. The model retained the same covariates as the primary fixed-effect

logistic regression (PC1, age, sex, and recruitment site). Inference for the Firth model was based on penalized profile likelihood. The Firth estimate was modestly attenuated relative to the conventional maximum-likelihood estimate but remained similar in magnitude and direction (OR = 2.25, 95% penalized profile-likelihood CI 1.30–4.28, p = 0.003), compared with OR = 2.47 (95% CI 1.32–4.62, p = 0.005) in the primary model (Supplementary Table S14).

Supplementary Table S14. Sensitivity analysis of the association between PC1 and HM Glucose using Firth bias-reduced logistic regression.

Model	N	OR for PC1	95% CI	p
Primary fixed-effect logistic regression	96	2.47	[1.32, 4.62]	0.005
Firth bias-reduced logistic regression	96	2.25	[1.30, 4.28]	0.003

Both models included PC1, age, sex, and recruitment site. For the conventional logistic regression, Wald confidence intervals and p-values are reported. For the Firth model, confidence intervals and p-values were based on penalized profile likelihood. OR = odds ratio; CI = confidence interval.

References

1. Hsu, Y.S.; Chen, T.Y.; Wu, D.; Lin, C.M.; Juang, J.N.; Liu, W. Te. Screening of Obstructive Sleep Apnea in Patients Who Snore Using a Patch-Type Device with Electrocardiogram and 3-Axis Accelerometer. *Journal of Clinical Sleep Medicine* **2020**, *16*, 1149–1160, doi:10.5664/jcsm.8462.
